# A Review on Biomarkers for the Evaluation of Autoimmune Cholestatic Liver Diseases and Their Overlap Syndromes

**DOI:** 10.3389/fmmed.2022.914505

**Published:** 2022-07-08

**Authors:** Henry H. Nguyen, Marvin J. Fritzler, Mark G. Swain

**Affiliations:** ^1^ University of Calgary Liver Unit, Department of Medicine & Department of Microbiology, Immunology and Infectious Diseases, Snyder Institute for Chronic Diseases, Cumming School of Medicine, University of Calgary, Calgary, AB, Canada; ^2^ Department of Medicine, Snyder Institute for Chronic Diseases, Cumming School of Medicine, University of Calgary, Calgary, AB, Canada; ^3^ University of Calgary Liver Unit, Department of Medicine, Snyder Institute for Chronic Diseases, Cumming School of Medicine, University of Calgary, Calgary, AB, Canada

**Keywords:** primary biliary cholangitis, primary sclerosing cholangites, autoimmune hepatitis, overlap syndromes, autoantibodies, microbiome & dysbiosis

## Abstract

Autoimmune cholestatic liver disease includes both Primary Biliary Cholangitis (PBC) and Primary Sclerosing Cholangitis (PSC). Both conditions result in impairment of hepatic bile flow ultimately leading to chronic liver injury, liver fibrosis and eventually end stage cirrhosis. Early and accurate diagnosis are important for the risk stratification, follow up and management of these patients. The underlying pathogenesis of these conditions have not been completely resolved and poses a barrier for the development of new diagnostic and prognostics tools. Current research work suggests that the pathogenesis of autoimmune cholestatic liver disease results from environmental, genetic, and a large component of underlying immune dysfunction. While the current available serum biomarkers and imaging modalities showcases progression in precision medicine for the management of autoimmune cholestatic liver disease, development of new biomarkers are still an area of need in this field. In this review, we will discuss the current and emerging biomarkers in patients with PBC, PSC, and a special population that exhibit overlap syndrome with autoimmune hepatitis (AIH). The use of these biomarkers for diagnosis and prognosis of these patients will be reviewed through the lens of the current understanding of the complex immune pathophysiology of these conditions.

## Introduction

Autoimmune cholestatic liver diseases are typified by both Primary Biliary Cholangitis (PBC) and Primary Sclerosing Cholangitis (PSC). Both conditions result from chronic immune mediated injury to the liver biliary system that can drive liver fibrosis and ultimately end-stage liver disease or cirrhosis. Although there are no current definitive cures for either condition, an early diagnosis and subsequent follow up of such patients can help in the management of debilitating symptoms, complications of cirrhosis, and identify patients who are candidates for curative liver transplantation.

PBC is a condition characterized by T-cell mediated injury of the biliary epithelium that chronically impairs bile flow leading to liver fibrosis and cirrhosis (Moebius et al., 1990). Epidemiological data has shown this condition is rare, affecting up to 402 cases per million persons in one US study (Kim et al., 2000) and has a predilection for females in the age range of 40–70 years with extrahepatic features that include fatigue, pruritus, and sicca symptoms (dry eyes and mouth) (Kim et al., 2000; Kaplan and Gershwin, 2005; Hirschfield et al., 2018). Although the underlying cause of PBC is not yet fully defined, various gene loci relating to immune function and an increased risk amongst monozygotic twins suggests a genetic component (Underhill et al., 1992; Selmi et al., 2004; Juran et al., 2010). Environmental factors, aberrant immune responses, chemical exposure, infections and molecular mimicry have also been suggested to play a potential role in the pathogenesis of the PBC (Shimoda et al., 2000; Bruggraber et al., 2003; Selmi et al., 2003; Bogdanos et al., 2004). The role of autoantibodies, which are serologic hallmarks for PBC, will be discussed below in the context of the pathogenesis, prognosis, and diagnosis of PBC.

PSC is a condition that is also characterized by presumed immune-mediated injury of the bile ducts leading to ductal scarring and narrowing, cholestasis, and progressive liver injury (Angulo and Lindor, 1999). Epidemiological studies of PSC are diverse in their findings with one meta-analysis suggesting an incidence rate of 0.75–1.0 per 100,000 person years, affecting males more than females with a median age of 41 years (Molodecky et al., 2011). The pathogenesis of PSC is also not fully understood, but various factors have been implicated. Genetic risk loci in human leukocyte antigen resulting in aberrant immune responses, bacterial infection, perturbations in the gut microbiome, changes in gut permeability and various environmental exposures are implicated in patients with PSC and the commonly associated condition of Inflammatory Bowel Disease (IBD) (Eaton et al., 2015; Karlsen et al., 2017; Dyson et al., 2018; Little et al., 2020).

The diagnosis of patients with either PBC or PSC requires serum biochemistry, serological biomarkers, imaging, and in certain clinical scenarios, liver biopsy. Although PBC and PSC are distinct autoimmune entities, there are patients who interestingly harbor overlapping features with autoimmune hepatitis (AIH) and are deemed to have either PBC-AIH or PSC-AIH overlap syndrome (OS). Detailed diagnostic and management strategies for OS are reviewed elsewhere (Boberg et al., 2011).

In this focused review, we highlight the currently available and emerging biomarkers of PBC, PSC and OS. These biomarkers will be reviewed in terms of their performance in the diagnosis and prognosis of these conditions. The different laboratory methodologies of testing for these autoantibodies and their performance in the clinical setting have been reviewed prior (van Beers et al., 2022). A summary of the autoantibodies with respective antigen targets and clinical utility can be found in Table 1.

**TABLE 1:** Summary of autoantibodies, the respective target antigens, and clinical utility in autoimmune cholestatic liver conditions

Antibody	Target antigen	Liver condition	Clinical utility
Anti-mitochondrial Ab (AMA)	Components of oxidative phosphorylation chain (i.e., PDC-E2, KGDC)	PBC	Diagnosis of PBC in combination with cholestatic liver enzymes
Anti-gp210	Transmembrane nuclear pore complex	PBC	Diagnosis of AMA negative PBC. Some studies correlate presence with poor prognosis
Anti-Tpr	Nuclear basket inner membrane of nucleus	PBC	Associated with PBC and other autoimmune conditions
Anti-centromere Ab	Centromere-kinetochore protein complex	PBC	Associated with PBC and other autoimmune conditions
Anti PML, sp100, sp140	Domains within the nuclear matrix	PBC	Diagnosis of AMA negative PBC. Some studies correlate presence with poor prognosis
Anti Hexokinase	Hexokinase located in outer membrane of mitochondria	PBC	Associated with both AMA positive and AMA negative PBC not yet validated for prognostication
Anti-Kelch-like 12	Collagen export and ubiquitination nuclear protein	PBC	Associated with both AMA positive and AMA negative PBC not yet validated for prognostication
Antineutrophil Cytoplasmic Ab	Cytoplasmic and perinuclear proteins of neutrophils and monocyte lysosomes	PSC	Limited diagnostic and prognostic use
Immunoglobulin G4	Various antigens are described with different organ involvement	IgG4-related dissases	Elevated serum levels with imaging and histology are diagnostic for IgG4 related disease

## Primary Biliary Cholangitis

### Anti-Mitochondrial Antibodies

The presence of specific anti-mitochondrial antibodies (AMA) in combination with cholestatic liver biochemistry (elevated serum alkaline phosphatase and gamma glutamyl transferase) are hallmark features and diagnostic of PBC. AMA have been thoroughly evaluated in the setting of PBC with high diagnostic sensitivity and specificity estimated at ∼85 and 98% respectively (Kaplan and Gershwin, 2005; Hu et al., 2014a). AMA target various components of the oxidative phosphorylation chain within the mitochondria, with the most common autoantibody target being the E2 component of the Pyruvate Dehydrogenase Complex (PDC-E2). Other targets of AMA, include the ketoglutaric acid dehydrogenase complex, branched chain 2-oxo-acid dehydrogenase complex and the dihydrolipoamide dehydrogenase-binding protein (Kaplan and Gershwin, 2005).

Presence of AMA is a highly useful diagnostic test given its specificity; however, using conventional immunoassays, the autoantibody titers do not reflect underlying disease activity. Current practice guidelines suggest treatment of PBC with ursodeoxycholic acid until normalization of cholestatic serum biochemistry is observed (Hirschfield et al., 2018), which has been shown to attenuate disease progression and significantly decrease the need for liver transplantation or death (Poupon et al., 1994; Harms et al., 2020; John et al., 2021). Accordingly, in practice, AMA titers are not serially monitored given the lack of association with disease severity and response to therapy.

### Anti-gp210 or Anti-Nuclear Rim Antibody

Anti-gp210 is a specific (>98%) anti-nuclear antibody targeting a transmembrane nuclear pore complex and can be diagnostic in PBC patients who do not have AMA (Courvalin and Worman, 1997; Haldar et al., 2021). Anti-gp210, unlike AMA, in patients with PBC has been associated with disease severity and poor prognosis in some studies (Muratori et al., 2003; Invernizzi et al., 2005), but not others (Stinton et al., 2011). Studies have shown mortality due to PBC associated hepatic failure/liver transplantation, clinical progression to hepatic failure, and histological evidence of severe interface hepatitis and lobular injury in patients harboring anti-gp210 (Invernizzi et al., 2001; Nakamura et al., 2007). In a single center study, in addition to adverse clinical outcomes, the presence of anti-gp210 was also associated with non-response to ursodeoxycholic acid (Haldar et al., 2021). Although not routinely used for monitoring, one study has suggested that a decrease in titer or loss of anti-gp210 antibody is associated with improved outcomes in PBC (Nakamura et al., 2005).

Anti-translocator promoter region antibodies (anti-Tpr) target a component of the nuclear basket adjacent to the inner membrane of the nucleus which functions to export messenger RNA (Enarson et al., 2004). Although anti-Tpr has been identified in the sera of patients with PBC (Ou et al., 2004), this autoantibody has not been evaluated in a larger cohort of patients with PBC and as such its performance in terms of diagnostic sensitivity, specificity and overall prognostication of PBC has not been validated. Of note, anti-Tpr has also been described in the sera from patients with systemic lupus erythematosus, dermatomyositis, autoimmune neuropathy, antiphospholipid syndrome, and arthritis (Ou et al., 2004).

### Anti-Centromere Ab

Classified as an anti-nuclear antibody, anti-centromere Ab targets the centromere-kinetochore macromolecular complex in patients afflicted with a variety of autoimmune conditions, primarily limited cutaneous systemic sclerosis, but also Sjögren syndrome, PBC (Kajio et al., 2021), and other autoimmune diseases (Fritzler et al., 2011). Although not used in isolation for the diagnosis of PBC, ACA was reported to be associated with disease progression, which included outcomes of both hepatocellular carcinoma and esophageal varices, in the absence of elevated serum bilirubin (Nakamura et al., 2007). ACA has also been shown in one limited study of 37 patients to be associated with increased risk of progressive annual decrease in glomerular filtration rate and overall chronic kidney disease (Mandai et al., 2013). Given the association of ACA with systemic autoimmune conditions and renal complications being uncommon in isolated PBC, this raises the question whether this finding reflects concurrent systemic illness rather than a specific feature of PBC itself.

### Promyelocytic Leukemia Protein Targets PML, Anti-sp100, and Anti-sp140

Anti-PML, anti-Sp100, and anti-sp140 target components of a dynamic structure referred to as PML nuclear bodies (Guion et al., 2019). These autoantibodies are also specific for PBC and are diagnostically useful in the setting of negative AMA serology (Muratori et al., 2009a; Hu et al., 2014b). From a prognostication standpoint, a decline of anti-Sp100 titers has been shown to be correlated with improvement in Mayo clinical risk score and response to ursodeoxycholic acid therapy (Mytilinaiou et al., 2012; Gatselis et al., 2013). Similarly, it has also been reported that anti-Sp100 or anti-PML titer is associated with more advanced liver histological staging and higher serum biochemistry (Bauer et al., 2021). As it stands, anti-Sp100 may be helpful for the diagnosis of PBC, however its utility for prognostication or defining a unique clinical phenotype remains unclear. The same can be stated about anti-sp140, as this marker is present in PBC but is not associated with a particular clinical course or outcome in this population (Granito et al., 2010).

### Anti Hexokinase 1 and Kelch-Like 12

Anti-HK1 targets an enzyme in the outer mitochondrial membrane that is involved in glucose metabolism and apoptosis. Anti-KLHL12, on the other hand, targets a nuclear protein implicated in collagen export and ubiquitination (Norman et al., 2015). Both anti HK1 and anti-KLHL12 have been shown to be more prevalent in PBC patients and in one study they had higher specificity than anti-gp210 and anti-Sp100 (Norman et al., 2015). These markers may also prove to be useful in PBC patients that do not harbor traditional autoantibodies such as AMA. Both anti-HK1 and anti-KLHL12 are present in ∼37.5 and 40% of PBC patients who are negative for AMA alone, or are combined AMA, gp210 and Sp100 negative, respectively (Reig et al., 2020). In terms of prognostication, the presence of anti-HK1 was associated with a significant reduction in transplant free survival and time to liver decompensation. Anti-KLHL12 has not been shown to be associated with adverse outcomes in terms of survival or progressive liver decompensation (Reig et al., 2020).

## Primary Sclerosing Cholangitis

Unlike PBC, the diagnosis of PSC is dependent on the presence of elevated cholestatic liver enzymes and magnetic resonance imaging findings of biliary structuring, as symptoms are often absent in early disease (Chapman et al., 2019). Reliable diagnostic and prognostic biomarkers are currently an area of unmet need in this condition. Current existing biomarkers are suboptimal in their performance and PSC patients harbor various non-specific autoantibodies, including anti-nuclear antibodies, anti-cardiolipin, and rheumatoid factor (Angulo et al., 2000).

### Antineutrophil Cytoplasmic Antibodies

The target of these autoantibodies are specific proteins in the cytoplasm of neutrophils and lysosomes of monocytes, primarily myeloperoxidase and proteinase 3, which are typically associated with a staining pattern that is cytoplasmic (C-ANCA for anti-PR3) or perinuclear (P-ANCA for anti-MPO) (Weiner and Segelmark, 2016). The evaluation of ANCA in PSC has shown that upwards of 84% of patients harbor this antibody (Angulo et al., 2000). Although anti-PR3 was previously reported to be present in a higher proportion of PSC patients compared to other autoimmune liver conditions (Stinton et al., 2014), its lack of specificity for PSC (Mulder et al., 1993; Weiner and Segelmark, 2016) limits its use in the diagnosis of PSC. The role of anti-PR3 has also been investigated as a potential biomarker for concurrent inflammatory bowel disease in patients with PSC, but not all studies have established a correlation (Stinton et al., 2014; Lee et al., 2019). There is evidence that the association of anti-PR3 with IBD/PSC may be related to the specific immunoassay used (e.g., ELISA versus chemiluminescence) and the possibility that novel epitopes of the target PR3 in PSC/IBD may not be available for autoantibody binding in ELISA (Stinton et al., 2014).

ANCA use is also limited for the management of patients with PSC from a disease monitoring standpoint. However, one recent study evaluated and subsequently validated ANCA (PR3) in combination with anti-GP2 IgA in PSC patients of European descent. In this study both autoantibodies were found to be associated with worse liver function tests, higher MELD scores, lower transplant free survival, and higher risk of cholangiocarcinoma (Wunsch et al., 2021). Whether this will translate to regular use within the clinical setting and/or international consensus guidelines for PSC management remains to be seen.

### IgG4 Associated Sclerosing Cholangitis

IgG4 related autoimmune disease can include a sclerosing cholangitis-like picture and is a distinct entity from PSC. IgG4 related disease, is an immune mediated condition that can lead to fibrosis in a variety of organs, including the liver. IgG4 associated sclerosing cholangitis can present similarly to PSC with elevated cholestatic liver enzymes and bile duct strictures seen on imaging. On liver biopsy, IgG4 related sclerosing cholangitis can show evidence of IgG4 plasma cells infiltrates along with fibro-inflammatory nodules (Deshpande et al., 2009). IgG4 associated sclerosing cholangitis typically occurs in the setting of concurrent autoimmune pancreatitis that can often be misclassified as pancreatic malignancy on initial presentation (Perugino and Stone, 2020). Elevated IgG4 levels in and of itself is not pathogenic but rather reflects the underlying aberrant immune response involving both B and T lymphocyte cell compartments driving disease development (Perugino and Stone, 2020). CD4^+^ T cells, T follicular helper cells, B cells, and the expanded IgG4 producing plasma cells have all been suggested to play a role in the hepatic and extrahepatic pathogenesis of this condition. Although IgG4 is not pathogenic, high serum IgG4 titers can correlate with the extent of organ involvement at the time of diagnosis and risk of relapse post immunosuppression (Wallace et al., 2016; Perugino and Stone, 2020). Its use for disease monitoring however is limited as these titers do not decrease in response immunosuppression (Perugino and Stone, 2020).

PSC with elevated serum IgG4 levels has also been described but should be distinguished from other IgG4 associated diseases which are typically responsive to immunosuppression (Zhang and Stone, 2019). The presence of autoimmune pancreatic inflammation, suggestive findings on liver histology (abundance IgG4 plasma cells, storiform fibrosis, and obliterative phlebitis), and markedly raised serum IgG4 levels (greater than 4 times the upper limit of normal) are some of the features that can be used to differentiate IgG4 associated diseases (including sclerosing cholangitis) from PSC (Ghazale et al., 2008; Culver and Barnes, 2017; Manganis et al., 2020). However, biomarkers that can differentiate steroid responsive IgG4 associated diseases from PSC, for which there is currently no approved therapy, is still an area of unmet medical need.

## Overlap Syndrome

Overlap syndrome describes patients harboring diagnostic features of more than one autoimmune liver condition, although it commonly describes overlap of either PBC or PSC with autoimmune hepatitis (AIH); a condition characterized by hepatocellular liver injury, specific serum autoantibodies, and features of interface hepatitis and plasma cell infiltrates on liver histology (Manns et al., 2015). Standardized diagnostic criteria for identifying patients with OS are lacking. OS patients are more likely to have poor clinical outcomes and require additional immunosuppression (Chazouillères et al., 2006; Muratori et al., 2009b). Some studies have suggested that the diagnosis of PBC-AIH OS can be established if two of three criteria for both PBC (abnormal cholestatic liver enzymes, +AMA, and florid duct lesions on biopsy) and AIH (hepatocellular enzyme elevation, elevated IgG or anti-smooth muscle antibody, or lymphocytic piecemeal necrosis consistent with interface hepatitis on liver biopsy) are fulfilled (Chazouillères et al., 1998; European Association for the Study of the Liver, 2009). The simplified AIH score, which includes ANA, anti-smooth muscle antibody, anti-LKM-1, SLA, IgG, histology, and exclusion of viral infections, has previously been suggested as a potentially useful tool to help identify patients with PBC-AIH OS (Boberg et al., 2011). However, this tool was not developed for, or validated in, OS and as such guidelines do not recommend using it for this purpose (European Association for the Study of the Liver, 2017; Mack et al., 2020). The diagnosis of PSC-AIH OS faces similar barriers given both the lack of robust serum autoantibodies and reliance on non-invasive imaging of the bile ducts for the diagnosis of PSC alone. Given the lack of uniform diagnostic criteria, the true prevalence of OS in patients with PBC and/or PSC are unknown. Understandably, without a universal diagnostic consensus for OS, specific biomarkers for PBC-AIH and PSC-AIH OS are also currently an area of unmet need. Previous studies have evaluated various serum autoantibodies and suggested that anti-p53 and anti-double stranded DNA can be part of the OS patient serum profile (Muratori et al., 2009b; Himoto et al., 2012). However, these markers are not exclusively unique to PBC and in fact anti-dsDNA can be found in and is highly specific for SLE (Chang et al., 2020; Pisetsky and Lipsky, 2020). Our group previously interrogated the sera of patients with biopsy proven PBC-AIH OS for potential autoantibodies that can be associated with OS. Amongst a total of 14 classical and novel autoantibodies assessed, only anti-dsDNA detected using the Crithidia luciliae assay was significantly associated with PBC-AIH OS. When this anti-dsDNA assay was used in combination with elevated ALT and IgG, this was associated with OS with AUROC of 0.84 (Nguyen et al., 2018). With the current paucity in biomarkers for OS, ongoing mechanistic studies will provide insight into disease pathogenesis and immune dysfunction that can lead to the discovery of new biomarkers.

## Emerging Biomarkers

### The Gastrointestinal Microbiome and Primary Biliary Cholangitis

The gastrointestinal microbiome is a collection of microbes within our gastrointestinal tract that has been implicated in modulating host immune responses and imparting an effect on various systemic conditions. Proposed mechanisms for these microbial effects include direct interaction with host mucosa, microbial derived metabolites acting on host cells, molecular mimicry, and direct effect on host immune responses (Lynch and Pedersen, 2016; Fan and Pedersen, 2021). The liver is intimately linked to the gastrointestinal tract via the portal circulation, anatomically positioned to respond to various signals and potential microbial perturbations in the gastrointestinal tract. Currently, it is unclear clinically whether the microbiome is simply a biomarker of liver disease state vs. a true driver of disease pathogenesis (Acharya and Bajaj, 2019). Work in pre-clinical or animal studies, however, suggests that the microbiome imparts a direct effect on the host response and liver disease outcomes (Albillos et al., 2020).

In the setting of PBC, the presence of different autoantibodies for specific antigenic targets has led to various theories about the underlying pathophysiology. The mitochondrial and nuclear antigen immune targets in PBC are thought to be made accessible to the immune cells within the liver by their expression on biliary epithelium (Lleo et al., 2010). The underlying driver of this autoimmune reaction has been postulated to include local liver microbial infection, immune cross reactivity with similar microbial antigens, and potential chemical exposure in animal models (Harada et al., 2001; Leung et al., 2003; Abdulkarim et al., 2004; Bogdanos et al., 2004). Given the potential role of microbes in the pathogenesis of PBC, the gut microbiota has been a subject of interest in recent research work. Studies evaluating PBC patients (including patients with no exposure to therapy with ursodeoxycholic acid) found a reduction in microbial diversity, and an overall alteration in the abundance of specific microbe genera vs. healthy controls (Lv et al., 2016; Tang et al., 2018). Interestingly, one study found that treatment of patients with ursodeoxycholic acid, which leads to clinical improvement in most patients, partially reversed some of these microbial population changes (Tang et al., 2018). A recent study by the same group further suggested that treatment of pruritus, a debilitating symptom in cholestatic liver disease, with a bile acid sequestrant was linked with compositional and functional differences in the host microbiota as well (Li et al., 2021). This potential link between autoantibodies, PBC development, and the microbiome is supported by other studies suggesting some similarities in pyruvate dehydrogenase complex E2, the dominant immune target in PBC, in humans and microbes. Interestingly, these studies also demonstrated cross-reactivity of PBC patient sera with components of *E. coli* and *N. aromaticivorans*, suggesting that microbial mimicry may be a potential initiating event in PBC pathogenesis (Selmi et al., 2003; Tanaka et al., 2018). Overall, however, the role of microbes in the pathogenesis of PBC is a work in progress, and additional studies are required to better evaluate the cause and effect of the host microbiome in PBC.

### The Gastrointestinal Microbiome and Primary Sclerosing Cholangitis

The association between PSC and IBD has made investigating the microbiome an area of great interest. A disruption of the microbiota homeostasis in IBD, termed dysbiosis, has been associated with host intestinal mucosal barrier changes, aberrant host immune responses, and luminal pathogen invasion that can culminate in chronic bowel injury (Caruso et al., 2020). IBD associated dysbiosis has also been implicated in shaping host bile acid and metabolite composition in the gut, which in turn can drive aberrant immune responses within the gut-liver axis in PSC patients. The various mechanistic means by which dysbiosis can potentially mediate downstream liver injury in PSC has been reviewed in detail previously (Shah et al., 2020). Like PBC, there have been association studies that have evaluated the microbiota composition in PSC patients and have highlighted a decrease in bacterial diversity, bacterial derived metabolites, and enrichment in specific taxa vs. healthy controls. Specifically, microbes reportedly enriched in the setting of PSC include *Streptococcus*, *Enterococcus*, and Veillonella species (Quraishi et al., 2019; Rühlemann et al., 2019; Lemoinne et al., 2020; Quraishi et al., 2020). Although definitive studies demonstrating the causation of microbiota in PSC patients have not been reported, pre-clinical studies have suggested that an altered gut-liver axis and the resultant changes in the liver immune environment are drivers of autoimmune cholestatic liver injury (Liao et al., 2019; Nakamoto et al., 2019). This expanding area of research will help to better define microbial or microbial-dependent host immune signatures that may be used to better diagnose and prognosticate patients with PSC.

An understanding of the complex role of the aberrant host immune response in autoimmune cholestatic liver disease will also potentially allow for identification of new biomarkers. Although a complete characterization of the immune cellular landscape and cytokine milieu in the liver of patients with autoimmune cholestatic disease is still needed, various inflammatory cytokines and immune cell populations have been implicated in autoimmune cholestatic liver disease (Banales et al., 2019; Yan et al., 2020). Furthermore, evaluating this altered liver immune and cytokine constellation through the lens of microbiota will be of importance with microbial and host immune interactions being implicated in inflammatory disease outcomes (Zheng et al., 2020; Balraj Singh et al., 2021).

## Conclusion

Autoimmune cholestatic liver disease includes PBC, PSC, and OS. Of the three conditions, PBC is the only one with reliable and specific diagnostic serum biomarkers. Both PSC and OS lack such a biomarker, with ongoing reliance on imaging and invasive liver biopsy to establish a diagnosis more confidently. Ongoing areas of unmet need in all three conditions include biomarkers to prognosticate disease progression and identify complications stemming from fibrosis. Furthermore, biomarkers that can complement our current use of serum biochemistry and bedside transient elastography to assess response to therapy are also needed. As we further understand the complexity of disease initiation and progression in autoimmune cholestasis, it is likely that a combination of patient variables and different serum biomarkers (including autoantibodies) and microbiome signatures will guide our care for this patient population.
